# Enhanced Mechanical Properties by Ionomeric Complexation in Interpenetrating Network Hydrogels of Hydrolyzed Poly (N-vinyl Formamide) and Polyacrylamide

**DOI:** 10.3390/gels7030080

**Published:** 2021-06-29

**Authors:** Joseph M. Scalet, Tiffany C. Suekama, Jeayoung Jeong, Stevin H. Gehrke

**Affiliations:** Department of Chemical and Petroleum Engineering, University of Kansas, Lawrence, KS 66045, USA; jmscalet@ku.edu (J.M.S.); tsuekama@gmail.com (T.C.S.); yg03077@ku.edu (J.J.)

**Keywords:** hydrogels, interpenetrating network, ionomer, polyelectrolyte, poly (N-vinyl formamide), polyacrylamide, polyvinylamine, poly (acrylic acid), modulus, toughness

## Abstract

Tough hydrogels were made by hydrolysis of a neutral interpenetrating network (IPN) of poly (N-vinyl formamide) PNVF and polyacrylamide (PAAm) networks to form an IPN of polyvinylamine (PVAm) and poly (acrylic acid) (PAAc) capable of intermolecular ionic complexation. Single network (SN) PAAm and SN PNVF have similar chemical structures, parameters and physical properties. The hypothesis was that starting with neutral IPN networks of isomeric monomers that hydrolyze to comparable extents under similar conditions would lead to formation of networks with minimal phase separation and maximize potential for charge–charge interactions of the networks. Sequential IPNs of both PNVF/PAAm and PAAm/PNVF were synthesized and were optically transparent, an indication of homogeneity at submicron length scales. Both IPNs were hydrolyzed in base to form PVAm/PAAc and PAAc/PVAm IPNs. These underwent ~5-fold or greater decrease in swelling at intermediate pH values (3–6), consistent with the hypothesis of intermolecular charge complexation, and as hypothesized, the globally neutral, charge-complexed gel states showed substantial increases in failure properties upon compression, including an order of magnitude increases in toughness when compared to their unhydrolyzed states or the swollen states at high or low pH values. There was no loss of mechanical performance upon repeated compression over 95% strain.

## 1. Introduction

Hydrogels can be crosslinked by either irreversible (covalent bonds) or reversible physical interactions (e.g., van der Waals interactions, ionic interactions, dipole-dipole interactions, and hydrogen bonding) [1,2,3]. Their ability to contain over 95% water content is a physical property that is desirable in many applications, particularly in the biomedical and pharmaceutical fields [4]. However, many hydrogels have poor mechanical properties, especially in terms of failure or fracture properties. A variety of strategies have been developed to toughen hydrogels [5,6]. Formation of double network (DN) hydrogels is the most widely recognized strategy for creating gels of superior mechanical properties, especially toughness. Double networks are IPNs made from two mechanically dissimilar gels. The first network is a rigid but brittle network, typically a polyelectrolyte, while the second network is soft and ductile [7,8]. When the DN is put under stress, the strain energy is dissipated by fracture of the covalent bonds in the brittle network and transfer of the stress to the ductile second network. As a result, under repeated stress loading cycles, the sacrificial bonds in the first network continue to break down with the second network gaining a greater influence on the mechanical properties of the IPN [9]. Thus, a limitation of DNs is that the first network’s sacrificial bonds are irreversibly broken [10], which may limit applications such as biomedical implants where repeated loading cycles may be applied. The development of self-healing hydrogels has been the subject of much recent interest [7,11]. Therefore, this work’s goal was to create a high charge density interpenetrating network of oppositely charged polyelectrolytes in which the ionic interactions would provide energy-dissipation mechanisms capable of self-healing [7,12,13,14].

The use of polymers with a high concentration of ionizable groups capable of forming ionic bonds, has been a topic of great interest in a large number of material science applications. These non-permanent links between the polymer chains are utilized as a means of energy dissipation to create high toughness gels [5,7,15,16]. For these materials, once a load that has disrupted the ionic bonds is removed, the ionic bonds can re-form, leading to a degree of self-healing in the hydrogel [17]. Previous work on poly (ethylene glycol)-based hydrogels have shown that inclusion of metal-ion complex to form ionic bonds between the polymer chains have greatly increased the toughness of the hydrogels [18]. Additionally, this same design motif of the self-healing ionic bonds has been shown to greatly toughen and improve the fatigue resistance of poly (acrylamide-*co*-acrylic acid) (PAAm-*co*-PAAc) and poly (vinyl alcohol) (PVA) hydrogels [19].

Several types of ionically complexed hydrogels have been described in the literature. One type is a polyelectrolyte complexed with a multivalent cation such as calcium alginate, as well as IPNs of calcium alginate and polyacrylamide [11,12,20,21,22,23,24]. Polyampholyte SNs are typically made by copolymerization of two oppositely charged monomers forming a network with both cationic and anionic groups [11,12,22,25]. A polyion complex gel can be made by the polymerization of a charged monomer in the presence of an oppositely charged polymer [12,14,26]. Some of these ionically crosslinked gels may have a significant degree of phase separation inside the material, or a low number of charged groups per molar mass of repeat unit, which can lead to fewer effective ionic bonds between the polymer chains [21]. Additionally, several examples of other ion complexed gels have been reported utilizing chitosan and gradually changing the pH to induce a charge in the chitosan [27,28].

When designing such ionically complexed polymers, there are many more options for anionic polymers than cationic polymers. A high charge-density cationic polymer is polyvinylamine (PVAm); it has one amine group per two backbone methylene groups. The vinylamine monomer is unstable, so PVAm cannot be made from the monomer, but it can be formed by hydrolysis of a polymer such as poly (N-vinyl formamide). We had previously formed PNVF hydrogels by copolymerizing in aqueous solution NVF monomer with a novel cross-linker, 2-(N-vinylformamido) ethyl ether (NVEE). N-vinyl formamide is an isomer of acrylamide (AAm), and this work showed that equivalent formulation of PNVF and PAAm had very similar mechanical and swelling properties in water. Both hydrogels had very similar χ parameters of about 0.5, varying modestly with polymer concentration [29]. Upon hydrolysis, both networks can form high charge density networks, with one ionizable group per two backbone methylene units, if complete hydrolysis were achieved. Hydrolysis of an amide group creates a carboxylic acid group with a pK_a_ of 4.5, and thus hydrolyzed polyacrylamide is negatively charged at higher pH values. In contrast, hydrolysis of a formamide group creates a primary amine group with a pK_a_ of 8.0, and thus hydrolyzed poly (N-vinyl formamide) can be protonated and become positively charged at lower pH values.

Due to the similar χ-parameters and the fact that the monomers of PAAm and PNVF are isomers, limited phase separation in a sequential IPN of the two networks is hypothesized. Both polymers can be hydrolyzed at similar rates to similar extents in basic solutions and thus were expected to undergo similar degrees of hydrolysis within an IPN of the two polymers [30]. Therefore, it was hypothesized that after hydrolysis of a neutral sequential IPN of PAAm and PNVF to form PAAc and PVAm (in the limit of complete hydrolysis), both the carboxyl groups on the PAAc and the amine groups on the PVAm would both be at least partially ionized and generate comparable amounts of positive and negative charge within the range of pH 4–8 [31]. This could enable a globally neutral gel to form via ion complexation. At pH values outside this range, the net network charge becomes unbalanced due to one of the two networks becoming deionized and the IPN would behave as a typical polyelectrolyte gel. Within this range, the IPN should deswell due to the loss of the osmotic swelling pressure of the mobile counterions. Additionally, we hypothesized that ionic bonds in the IPN within this pH range would lead to a gel with superior material properties [7,32]. In principle, acrylic acid could be used to form the anionic network, but in most formulations, this would lead to an IPN with a net negative charge in most above the pK_a_ since the counterpart PVAm hydrolysis does not proceed to completion.

In summary, the synthesis of sequential PAAm/PNVF and PNVF/PAAm IPNs followed by simultaneous hydrolysis was hypothesized to lead an IPN of both positively and negatively charged networks which would to collapse at intermediate pH vales and exhibit improved failure properties due to charge complexation. To the best of our knowledge, this would be the first reported synthesis of an IPN with ionic complexation created from two neutral, thermodynamically compatible networks with the potential to reach a high number of ionic bonds.

## 2. Results

The polyelectrolyte IPN hydrogels were synthesized from a sequential IPN of two neutral networks. While the neutral networks showed only slight improvement in mechanical properties over a single network of the same polymer concentration at synthesis, the properties of the hydrolyzed IPNs were considerably different from the unhydrolyzed IPNs. At intermediate pH values, the hydrolyzed networks showed a drastic decrease in swelling, increase in modulus, and a much higher fracture stresses and strains. These changes in properties gave rise to a much tougher hydrogel than a comparable single network hydrogel and support the hypothesis of reversible ionic bonds forming between the two oppositely charged networks.

The IPNs were formed sequentially, synthesizing the first network, then soaking the first network in a solution of the second monomer, followed by initiation of polymerization to form the second network. Both combinations of the two polymers were made: PNVF followed by PAAm and PAAm followed by PNVF. The mass of the network was measured after the first network was formed and again after the second network was created. Based on the change of mass it was found that the composition of the networks was 48:52 for PAA/PNVF hydrogels and 55:45 for PNVF/PAAm. The subsequent hydrolysis of the IPNs was expected to plateau at around 40% to 50% conversion [33].

**Figure 1:**
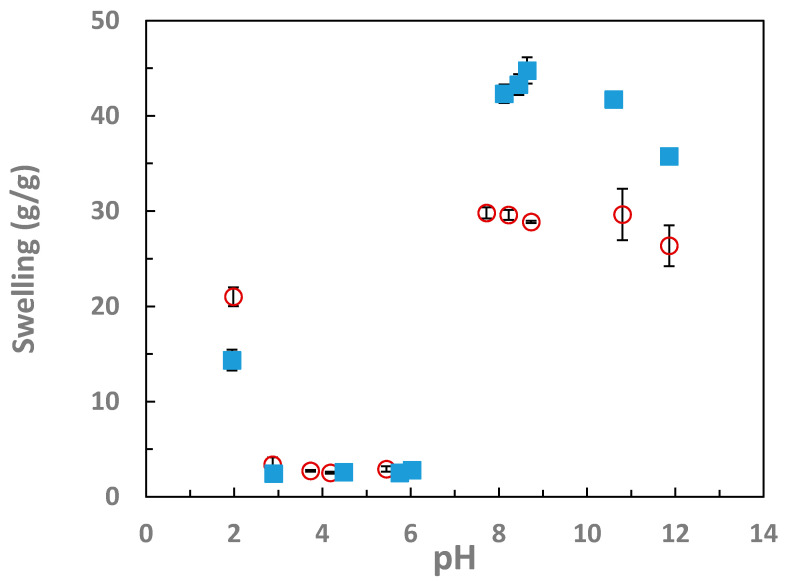
IPN swelling degree as a function of solution pH. The sharp decrease in swelling between pH 3 and 6 is consistent with charge complexation of the two networks. IPNs of PAAm/PNVF and PNVF/PAAm were partially hydrolyzed to PVAm/PAAc (blue closed symbols) and PAAc/PVAm (red open symbols).

The equilibrium swelling degree of the partially hydrolyzed IPNs of PVAm/PAAc and PAAc/PVAm were plotted against the pH, as shown in Figure 1. After hydrolysis, the swelling degree was 18.42 ± 0.03 for PAAc/PVAm and 23.03 ± 0.47 for PVAm/PAAc in 0.1N NaOH. After leaching out the base, these gels were then equilibrated in solutions of pH from 2 to pH of 6. The gels collapsed at these intermediate pH values from 3 to 6 with a swelling degree of 3 on average. When at pH of 2, the gel swelled to 14.35 ± 1.10 for PAAc/PVAm and 21.01 ± 0.99 for PVAm/PAAc. At higher pH (8–12), the swelling degree is around 30 for PAAc/PVAm and approximately 40 for PVAm/PAAc. The PVAm/PAAc IPN shows a decrease in swelling degree at above a pH of 10 (Figure 1). At higher pH, the swollen volume increases until a maximum is reached and then decreases, due to the increasing ionic strength, which is conventional polyelectrolyte gel behavior [34]. It should be noted that in this work that only the pH of the solution is measured, while the pH inside the gel can differ due to the Donnan equilibrium effect creating an unequal ion exchange [35]. This can cause the expected behavior of the gel based on pK_a_ to be shifted relative to the solution pH [32].

**Figure 2:**
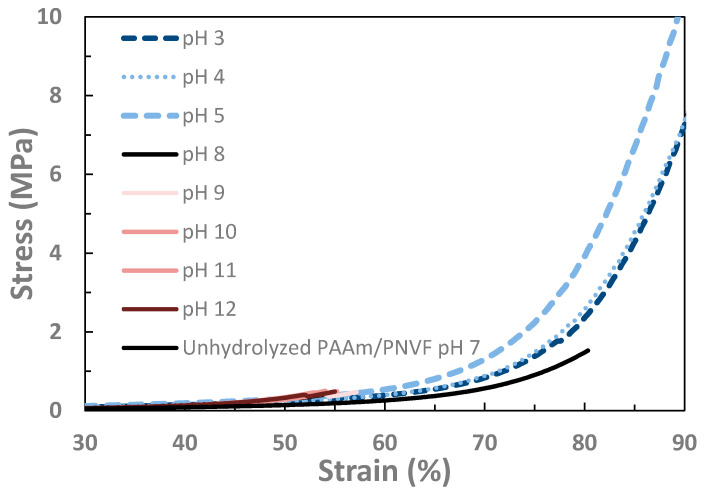
Representative stress–strain curves for partially hydrolyzed PAAc/PVAm IPNs at various solution pH values. At pH values between 3 and 6 the fracture stress and strain increase substantially with a sharp upturn at high strain values relative to both the unhydrolyzed IPN (solid black line) and the hydrolyzed networks outside this pH range (curves at pH values of 9–12) drawn in shades of red are shown to nearly overlap on this scale prior to fracture around 55%). This is consistent with the hypothesized behavior for an IPN with ion complexation between the component networks. Note that gels at pH 3, 4, and 5 did not fracture until strains over 95%, but the data shown are truncated at 90% due to the limited accuracy of compressive testing at such high strains.

The mechanical properties of the hydrogels were evaluated by uniaxial compression in order to measure their failure properties, modulus, and toughness (work to failure). Representative curves are shown in Figure 2. The unhydrolyzed IPN has a fracture strain over 80%, but with a relatively low fracture stress, modulus and toughness. After hydrolysis, at solution pH values of 3, 4 and 5 all of the values increased substantially: in fact, these gels did not fracture even above 95% compressive strain. The performance is highest at a solution pH of 5, where it is likely that a maximum degree of ionic bonding would be observed. In contrast, the hydrolyzed gels outside this range are quite brittle and fracture below 55% strain.

**Figure 3:**
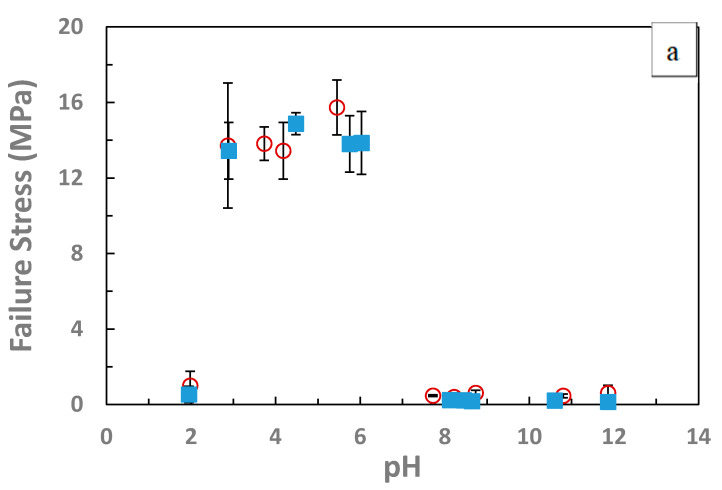

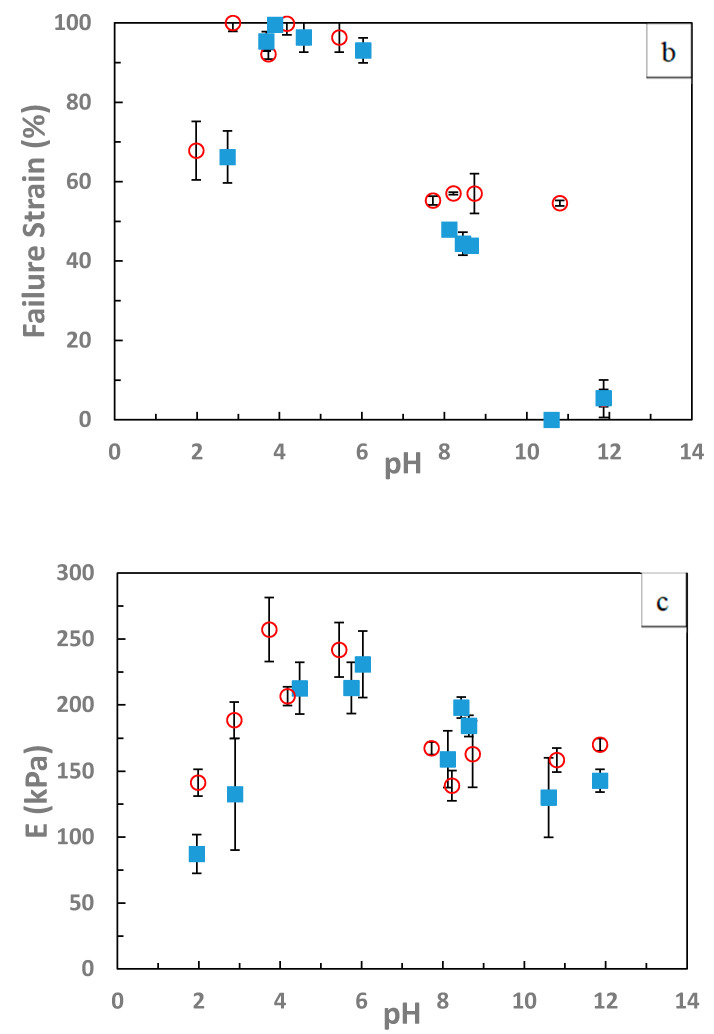
(**a**–**c**): Changes in mechanical properties with solution pH. (**a**) Ionically complexed IPNs show large improvements in failure stress and failure strain at intermediate pH, the failure properties are improved in comparison to the uncomplexed state. IPNs of PAAm/PNVF and PNVF/PAAm were partially hydrolyzed to PVAm/PAAc (blue closed symbols) and PAAc/PVAm (red open symbols).

**Figure 4:**
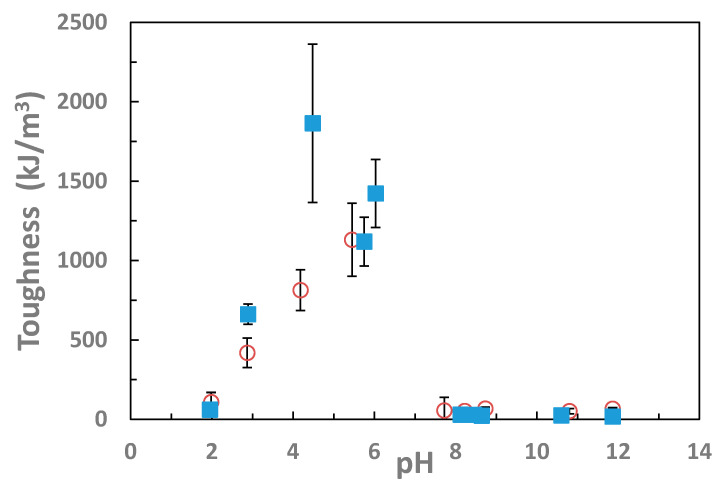
Hydrolyzed IPN toughness increased at intermediate pH values. The toughness at the intermediate pH is much greater than at the uncomplexed state. IPNs of PAAm/PNVF and PNVF/PAAm were partially hydrolyzed to PVAm/PAAc (blue closed symbols) and PAAc/PVAm (red open symbols).

The full set of physical property data obtained from the stress–strain measurements are plotted in Figure 3 and Figure 4 and tabulated in Table 1 and Table 2. Both sets of IPNs had improved failure stress and failure strain at the intermediate pH values, as shown in Figure 3. The failure strains at the intermediate pH (3–7) were all virtually 100% while the failure stresses are about 14 MPa. The PVAm/PAAm IPN treated with 0.1 M HCl, and then water has a failure stress that is slightly lower than 14 MPa.

The failure stress of the complexed state seen in Figure 3 a of ~15 MPa is comparable to that reported in the classic work of Gong et al. which showed IPN of poly (2-acrylamido-2-methylpropanesulfonic acid) (PAMPS) and PAAm to have a failure stress of 17.2 MPa. This work showed increases in the failure stress of the hydrogel for each of the single networks hydrogels from 0.4 MPa and 0.8 MPa to 17.2 MPa when the two were combined in a double network, comparable to the changes seen in Figure 3a from uncomplexed to complexed IPNs [36]. Comparable changes in the failure stress are also seen in the hydrolyzed IPNs between the uncomplexed state and the complexed state. The values are also comparable to those reported by Luo et al. for the polyion complexes in compression of a fracture stress of 17.5 MPA and strains of 95% without fracture, and work of extension in tension of 14.8 MJ/m^3^.

Figure 4 depicts that the work to failure toughness of the IPNs and follows the general trends from the failure stress and failure strain data in Figure 3a,b. At the intermediate pH, both the IPN gels’ toughness is greatly improved relative to the un-complexed state. From pH 3 to pH 6 there is a slight positive trend in toughness, which is consistent with the increased ionization of the PAAc network as the pH increases past its pK_a_ value of 4.5. However, once the pH reaches values above 8.0, the PVAm network, which has a pK_a_ of 8.0, will diminish in ionization until it becomes neutral. The PAAc/PVAm IPN behavior is quite similar, though it has a slightly higher toughness than the corresponding PVAm/PAAc in all conditions.

To test whether the neo-Hookean assumption is valid for this system, Mooney–Rivlin plots were generated. It was found that the Mooney–Rivlin linearization plots of the hydrogels in the collapsed state were nearly horizontal with C_2_ values near 0 between 15% to 50%. Along with measuring an E/G ratio of 3, this indicates that these materials behaved as neo-Hookean solids in this range.

However, as the strain exceeded 50%, there was a significant upturn in both the Mooney–Rivlin and stress–strain plots (Figure 2), deviating from neo-Hookean behavior. As the pH increased, the C_2_ of the Mooney–Rivlin plot also increased from 0.1 ± 2.0 at pH 3.0 to 14.4 ± 2.3 at pH 12.0. Additionally, the C_1_ values decreased as the pH increased, leading to a difference in the shear modulus G when calculated from the stress–strain plot vs. the Mooney–Rivlin plots. All of this indicates that at higher pH, unlike the complexed states, the net charged states behave as Mooney–Rivlin solids with positive C_1_.

**Figure 5:**
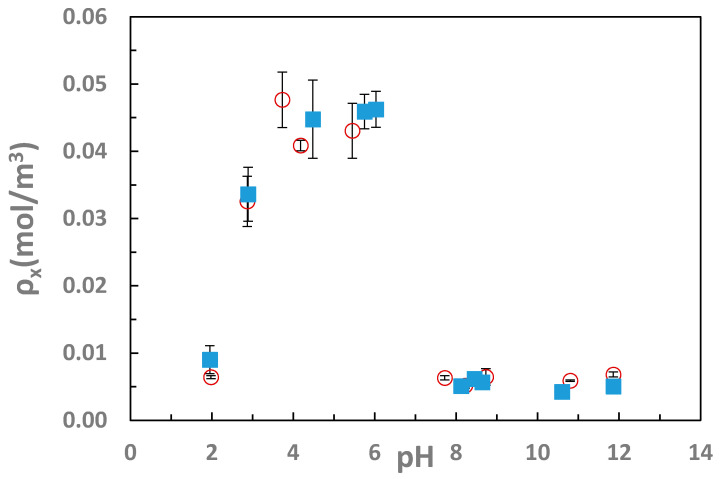
Crosslink density as a function of pH. A drastic difference between the collapsed state between pH 3 and 6 can be seen compared to pH values greater than eight or less than 2.

At the low to intermediate pH values where the hydrogels were collapsed, the toughness was calculated at 90% strain rather than at failure due to limits of compression testing with the instrument, even though many of these gels did not fail until virtually 100% compression, so the toughness and failure properties reported in Table 1 and Table 2, as well as Figure 4 are the lower limits on these failures. However, an effective crosslink density was calculated from shear modulus assuming the affine model of rubber elasticity [37], shown in Figure 5, from the swelling and mechanical testing, as shown in the Table 1 and Table 2. While the decrease in swelling is expected to increase shear modulus of an ideal elastomer without changing the crosslink density on a dry basis, the affine model modified for use with swollen gels takes both swelling and modulus into account. Figure 5 shows the large change in the crosslink density between the swollen states at both high and low pH values and the intermediate pH values. At both pH 2 and pH values above 7, the average crosslink density was calculated to be around 0.005 mol/m^3^, while in the collapsed state, the average crosslink density was nearly an order of magnitude higher, around 0.04 mol/m^3^.

**Figure 6:**
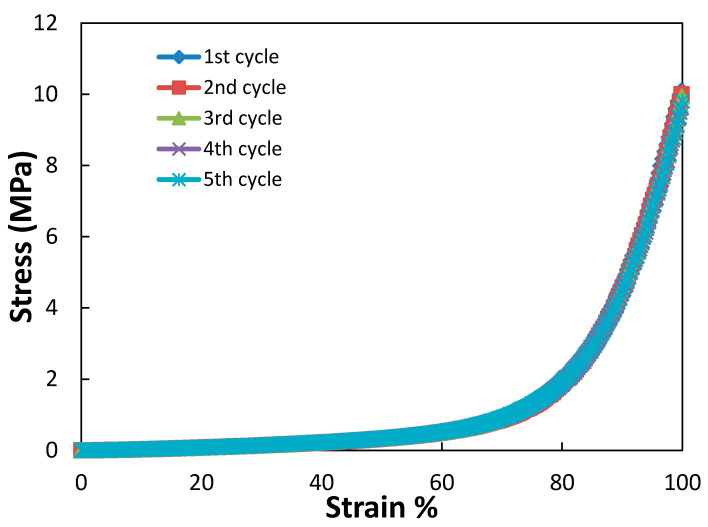
Repeated loading of a partially hydrolyzed PAA/PNVF hydrogel. After five high strain compression cycles there was negligible change in the stress–strain curves of the hydrogel, indicating the hydrogel did not undergo any irreversible damage due to repeated loading cycles.

Finally, a key hypothesis for this work was that ionic complexation in the IPN would enhance the mechanical performance of the IPN without deterioration of properties upon repeated cycles as seen in double networks and certain other tough gels [6,7]. Thus stress–strain curves of a single sample were measured on repeated loading and unloading cycles. As shown in Figure 6, there was no notable change in the stress strain-behavior over 5 compression cycles, consistent with the complexation hypothesis (though hysteresis was not measured).

## 3. Discussion

The motivation for this work was the development of a high toughness hydrogel that did not derive its toughness from sacrificial covalent bonds as in double networks [22]. Toward this end, it was desirable to create a hydrogel that achieved high toughness using self-healing ionic bonds [38]. While hydrogels with two oppositely charged groups have been synthesized previously [5,11,15,32,35,38], here, the creation of such a hydrogel starting from two thermodynamically compatible neutral networks has been demonstrated. In general, polyelectrolyte mixtures undergo a degree of microphase separation [21,22]. Starting with two neutral monomers that are isomers of each other and exhibit similar χ parameters as with NVF and AAm, an IPN hydrogel formed was optically clear, which would be expected when there is a high degree of intermolecular mixing at a sub-micron scale and limited phase separation. It was shown that the order of the two networks in the sequential IPN, whether P(NVF)/PAAm, PAAm/P(NVF) or the hydrolyzed PVAm/PAAc, PAAc/PVAm did not matter, as the amounts of the two networks was found to be approximately equal which was confirmed by mass analysis, and the swelling and mechanical properties were statistically similar. The consistency of the data also indicates that the extent of hydrolysis of both networks was nearly equal regardless of the ordering of the networks.

As expected of an IPN made from two similar networks, the properties of the unhydrolyzed hydrogel were similar though not identical to a previously reported equivalent single networks of the same mass composition of 30 wt% [29]. There was a modest increase in toughness in the IPNs relative to the SNs, with the single networks having a toughness of 90 ± 22 kJ/m^3^ and PAAm being 75 ± 20 kJ/m^3^ compared to the two IPNs which had an average of 163 ± 18 kJ/m^3^. Additionally, the shear modulus when comparing the unhydrolyzed IPNs to the 30 wt% single networks showed no significant difference with an average of 97 ± 6 kPa for the IPNs compared to 83 ± 18 kPa and 105 ± 74 kPa for PNVF and PAAm single networks, respectively.

**Figure 7:**
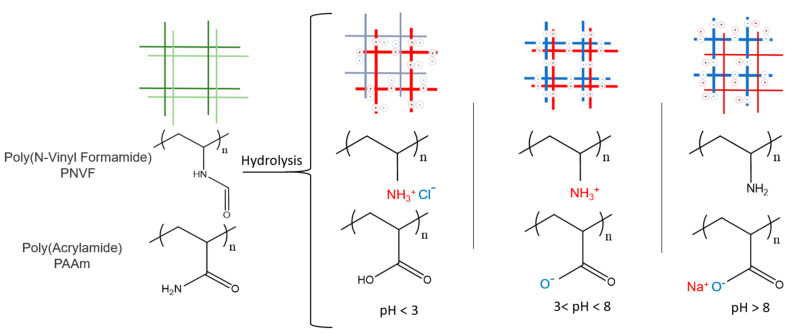
Formation and partial hydrolysis of PNVF and PAAm to PVAm and PAAc. When in an acidic solution with a pH of less then 3 the PVAm is ionized while the PAAc is not, leading to a highly swollen gel due to the osmotic pressure of the mobile counterions associated with the protonated amines. The reverse happens in a solution with a pH greater then 8, the PAAc is ionized with mobile cations while the PVAm is not. However, at intermediate pH values between 3 and 8 networks are both ionized, Thus the polymers can from ionic bonds with each other without mobile counterions. Thus in this state the two networks form a globally neutral hydrogel that deswells. These ionic bonds formed between the two networks lead to the increased mechanical properties reported.

However, after hydrolysis, physical properties changed significantly. At the higher pH values, the gels tended to be more brittle and have a higher swelling degree when compared to the unhydrolyzed IPN hydrogels, as shown in Figure 1 and Figure 3. A schematic of the interactions is provided in Figure 7. The increase in swelling in basic solution is due to the poly(vinyl amine) groups being in the free base form of R-NH_2_ while the poly(acrylic acid) are in the charged sodium salt form R-COO-Na+. This creates a difference in mobile ion concentration between the inside the gel and the solution, leading to a large increase in the osmotic swelling pressure, thus causing a highly swollen gel [34,39,40]. The hydrolyzed gels were also highly swollen when equilibrated with an acidic solution of pH 2; the swelling degree was 21 for PAAc/PVAm and 14 for PVAm/PAAc. At this pH, the vinylamine groups will be positively charged salt form of R-NH_3_^+^Cl^−^ and the acrylic acid groups will be in the neutral R-COOH form. In both cases, this is typical polyelectrolyte gel behavior. The ionic osmotic swelling pressure is reduced when sodium chloride is added to the solution at either high or low pH. This behavior was observed when the gels are placed in an NaCl solution of moderate concentration, causing the gels in basic solutions to de-swell significantly to only 15–20 instead of the 30–40 seen in water. Similar deswelling occurs with NaCl solution at acidic pH.

However, at intermediate pH values of 3–7, the swelling degree dramatically decreased to an average of 2.7 ± 0.2 between the two gels, and there was no significant effect on swelling with added salt. At this state, it is hypothesized that both the amine and the carboxylic acid are in their charged forms and forming complexes with each other via intermolecular ionic bonds. As seen in Figure 5, these complexes acted as additional effective crosslinks in the IPN over this stress–strain range [39,41,42]. This balance of counterions leads to a globally neutral gel with reduced mobile ion concentrations inside the gel leading to the low osmotic swelling pressure and the salt insensitivity observed. It should be noted that these are the pH values of the solutions, while the pH inside the gel can differ due to the Donnan equilibrium [35,43].

In this intermediate pH range, failure properties were greatly increased on the order of 3-fold increase in the failure stress and 30-fold for the toughness. As shown in Table 1 and Table 2 and Figure 2, Figure 3 and Figure 4, there is a significant difference in the mechanical properties between the intermediate pH complexed states and uncomplexed states at very low and high pH values. While not as dramatic a difference as in the changes in swelling degree there is a significant difference in the compressive modulus of the hydrogels between 4 and 6 pH and those with a pH greater than 9. Thus, when the gels are complexed and thus collapsed, the modulus increases, which indicates along with the decreased swelling that the networks have a greater concentration of effective crosslinks at these states. Additionally, in the stress–strain curves for the gels in Figure 2 there is a strong upturn in the graphs past 60% for the collapsed gels. This is the same region where the Mooney–Rivlin C_2_ value become strongly positive. This is theorized to be evidence of the ionic bonds between the networks dissipating strain energy upon this further deformation. The repeatability of the stress–strain behavior to compressive strains over 95% as shown in Figure 6 is also strong indication of the ability of the ionic bonds to self-heal once the load is removed.

## 4. Materials and Methods

### 4.1. Materials

The reagents for PNVF and PAAm networks are as follows [29]. N-vinyl formamide (NVF; Sigma-Aldrich, St. Louis, MO, USA: 98%) monomer was purified by distillation under vacuum at 80 °C and stored at −10 °C before polymerization. A novel cross-linker, 2-(N-vinyl formamide) ethyl ether (NVEE; liquid of density of 1.3 g/mL), was synthesized and characterized by our previously reported procedure [10,29,44]. The monomer, acrylamide (AAm; Sigma-Aldrich: 99+%) and the cross-linker, N,N’-methylenebisacrylamide (BIS; Sigma-Aldrich, St. Louis, MO: 99+%) are electrophoresis grade and were used as received. Hydrolysis was carried out using 0.1 M NaOH (Fisher Scientific, Pittsburgh, PA, USA). The initiator, 2,2′-Azobis[2 -(2-imidazolin-2-yl) propane] dihydrochloride (VA-044; Wako Pure Chemical Industries, Ltd., Osaka, Japan), was used as received. The different pH solutions were made by dilution of stock solutions of HCl (Fisher Scientific, Pittsburgh, PA) and of NaOH.

### 4.2. Synthesis of IPN Hydrogels

The conventional gel formulation notation, *T* × *C*, was used to describe the gel composition [45,46]. *T* represents the total mass of monomer and cross-linker over the volume of water in which it was dissolved (*w*/*v*) as a percentage:

(1) $$T=\frac{total\;mass\;\left(monomer+crosslinker\right)\left(g\right)}{Volume\;of\;water\;\left(ml\right)}*100$$

*C* represents the ratio of the mass of cross-linker by the total mass of monomer and cross-linker (*w*/*w*) as a percentage:

(2) $$C=\frac{mass\;of\;crosslinker\;\left(g\right)}{total\;mass\;\left(monomer+crosslinker\right)\left(g\right)}*100$$

Each network for this study was formulated using a 15 × 3 (*T* × *C)* formulation. Thus, PNVF synthesis is as follows: NVF (0.566 mL), NVEE (0.013 mL) and thermal initiator VA-044 (0.15% *w*/*v*) were added to 4 mL of DIUF water in a 5 mL glass vial. A similar procedure was used for the PAAm gel. PAAm gel was synthesized by dissolving AAm (0.582 g) and BIS (0.018 g) in 4 mL of DUIF water. This polymerization also used the VA-044 (0.15% *w*/*v*) as the initiator. The solution was then mixed in both formulations, bubbled with nitrogen for 15 min to displace dissolved oxygen, and then pipetted into molds consisting of two glass plates with a 2 mm height silicon spacer. The molds were wrapped with plastic wrap and clamped with binder clips to eliminate evaporation. The free radical polymerization was carried out at 50 °C in a preheated oven for 24 h. After the reaction was complete, a molded SN of PNVF or PAAm resulted.

To synthesize an IPN of PNVF/PAAm or PAAm/PNVF, the first network was soaked in a 15 wt% solution of the second network, using the same 15 × 3 formulation as noted above for ~ 48 h. The soaked gels are then placed between two glass plates, wrapped with plastic wrap, and placed in a 50 °C oven for 24 h. The resulting hydrogel is an IPN of PNVF/PAAm or PAAm/PNVF. Before hydrolysis, the IPNs were cut into cylindrical disks (~4 mm diameter and ~2 mm height) using a 4 mm biopsy punch.

### 4.3. Hydrolysis of Hydrogels

**Figure 8:**
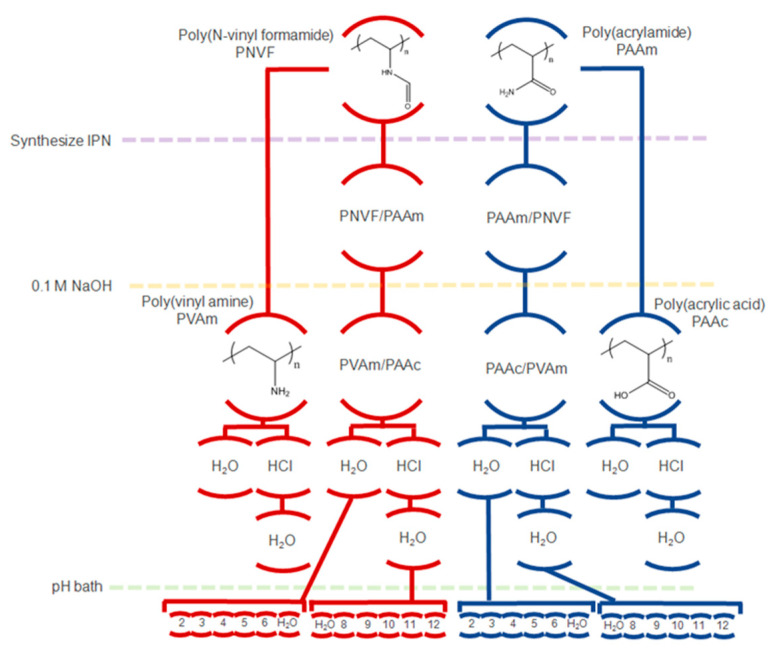
Schematic of formulations and the treatments.

Figure 8 shows a schematic of the types of hydrogels synthesized in this study and the treatments performed. Samples were made using both PNVF and PAAm as the starting network for the gel. After formation, the gels were soaked in a solution of the opposite monomer and allowed to equilibrate before the second network was polymerized. Once the IPN was formed, it was then hydrolyzed with 0.1 M NaOH to form the IPN of interest. Finally, the pH of the solution in which the IPN was in adjusted to the value of interest ranging from 2 up to 12.

The conditions to achieve hydrolysis with the mildest conditions feasible to avoid degradation of the network (low NaOH, low temperature, and shortest time) were determined from literature to be 0.1M NaOH at 60 °C for 24 h [30,47,48]. Cylindrical disks punched out by biopsy punch of PNVF, PAAm, PNVF/PAAm, and PAAm/PNVF hydrogels were hydrolyzed by placing them in a container of 0.1 M NaOH and then placing them in the oven at 60 °C for 24 h. The hydrolyzed gels were leached in water, changing the water multiple times to remove residual chemicals over 24 h. This leaves the carboxylic acid in the sodium salt form R-COO^−^Na^+^ and the amines in the free base form R-NH_2_. Half of the hydrogels were placed in 0.1M HCl, changing the HCl solution multiple times for 24 h and then placed back in the water (changing the water multiple times) to remove residual HCl, again for 24 h. Thus, the carboxylic acid groups will be in the non-ionized state R-COOH, and the amines are in the hydrochloride salt R-NH_3_^+^Cl^−^. 

The half of the hydrolyzed IPNs of PVAm/PAAc and PAAc/PVAm which were not treated with HCl were placed in pH baths of either a pH of 2, 3, 4, 5, 6 and the IPNs that were treated with HCl were placed in a pH bath of either a pH of 8, 9, 10, 11 or 12. The gels were soaked for 48 h, changing the pH bath multiple times. The pH of the solution of all of the baths was measured. On many occasions, the pH was different from the original bath solution (even at equilibrium) because the gel acts as a buffer; therefore, a pH probe’s final pH was taken. The reported data were correlated with this measured pH.

### 4.4. Swelling and Mechanical Tests

The procedure for swelling and the mechanical analysis were followed as previously described above [29]. The degrees of swelling, Q (g/g), is reported as the swollen gel’s mass over the mass of the desiccator-dried gel [29]. Unconfined, uniaxial compression was performed using an RSA III dynamic mechanical analyzer (TA Instruments) at a rate of 0.05 mm/s. The sample diameter was measured using a micrometer under a standard stereomicroscope (~10× magnification). The compression plates were protected by a nitrile film which was lubricated with mineral oil. The nitrile film protected the plates from harsh pH conditions but did not affect the mechanical test results as was confirmed by measuring the mechanical properties of the unhydrolyzed gel were the same both with and without the nitrile film. The stress–strain curves were evaluated to determine the mechanical properties as described in the results section above. Thus, the shear modulus G was found using strain values up to 60% and the compressive modulus, E, using strain values up to 15% using equations below.

(3) $$\sigma =\frac{F}{A_{0}}=G\left(\lambda -\lambda^{-2}\right)$$

(4) $$\lambda =\frac{L}{L_{0}}$$

(5) σ=Eε=E(λ−1)

From the shear modulus and swelling data, an effective crosslink density was calculated using the affine model adjusted for a network formed in solution and swollen to equilibrium, as given in Equation (6) [37].

(6) $$\rho_{x}=\frac{G}{RT\phi_{2}{\left(\phi_{2f}/\phi_{2}\right)}^{2/3}}$$

where *R* is the ideal gas constant; *T* is the temperature; $\phi_{2}$ is the polymer volume fraction in the gel at testing conditions; $\phi_{2f}$ is the polymer volume fraction at network formation; ρ_x_ is the effective cross-link density in the polymer network (mol/cm^3^). This model assumes that the chains have a Gaussian distribution of chain conformations, which is likely not valid in the highly swollen states. In principle, a correction for the non-Gaussian distribution under such conditions could be applied [49].

## 5. Conclusions

This report describes the novel synthesis of an polyelectrolyte IPN hydrogel derived from simultaneous hydrolysis two neutral networks is capable of ionic complexation. This IPN of ionizable PVAm and PAAc has greatly improved toughness and failure properties in comparison to unhydrolyzed PVNF and PAAm IPN hydrogels. The ionic complexation proposed can explain the rise to a dramatic decrease the swelling degree and increase in mechanical properties under compression at intermediate pH values. This resulted in a much tougher gel when compared to the equivalent nonionic IPN. Possible energy dissipation by disruption of ionic bonds is suggested by the sharp upturn in the stress at high strains and the consistency of the mechanical properties upon repeated high strain loading indicates the potential for self-healing of these bonds. As the IPNs did not undergo failure at the upper limits of compressive strain, it is expected that the toughness could be much higher in tension.

## Figures and Tables

**Table 1:** Mechanical Properties of PAAc/PVAm IPNs.

pH	Failure Stress (MPa)	E (kPa)	G (kPa)	E/G	Q (g/g)	ρ_x_ (mol/m^3^)
2	1.00 ± 0.76	140 ± 12	45 ± 3	3.15 ± 0.13	21.0 ± 0.8	0.0064 ± 0.0002
3	13.72 ± 3.30	190 ± 17	66 ± 4	2.86 ± 0.78	3.4 ± 0.6	0.0330 ± 0.0037
4	13.44 ± 1.50	210 ± 9	69 ± 2	3.97 ± 0.24	2.5 ± 0.1	0.0410 ± 0.0008
5	15.74 ± 1.50	240 ± 25	80 ± 9	3.01 ± 0.03	2.9 ± 0.2	0.0430 ± 0.0041
6	13.82 ± 0.89	210 ± 24	85 ± 8	2.50 ± 0.08	2.7 ± 0.1	0.0480 ± 0.0041
8	0.47 ± 0.46	260 ± 30	56 ± 2	4.63 ± 0.05	30.0 ± 0.5	0.0063 ± 0.0003
9	0.40 ± 0.11	170 ± 6	45 ± 4	3.68 ± 0.05	30.0 ± 0.4	0.0052 ± 0.0004
10	0.62 ± 0.14	140 ± 14	56 ± 13	2.49 ± 0.13	29.0 ± 0.1	0.0065 ± 0.0012
11	0.46 ± 0.98	160 ± 11	52 ± 5	3.05 ± 0.10	30.0 ± 2.2	0.0059 ± 0.0001
12	0.61 ± 0.42	170 ± 6	55 ± 1	3.08 ± 0.03	26.0 ± 1.7	0.0068 ± 0.0004

**Table 2:** Mechanical Properties of PVAm/PAAc IPNs.

pH	Failure Stress (MPa)	E (kPa)	G (kPa)	E/G	Q (g/g)	ρ_x_ (mol/m^3^)
2	0.54 ± 0.43	87 ± 18	45 ± 10	1.95 ± 0.83	14.0 ± 0.9	0.0090 ± 0.0021
3	13.44 ± 1.50	130 ± 52	52 ± 6	2.56 ± 0.78	2.5 ± 0.1	0.0340 ± 0.0040
4	14.88 ± 0.58	210 ± 24	71 ± 8	2.98 ± 0.02	2.6 ± 0.2	0.0450 ± 0.0058
5	13.81 ± 1.50	210 ± 24	72 ± 4	2.95 ± 0.16	2.5 ± 0.1	0.0460 ± 0.0026
6	13.86 ± 1.70	260 ± 30	78 ± 10	3.29 ± 0.03	2.8 ± 0.3	0.0460 ± 0.0027
8	0.18 ± 0.38	230 ± 31	60 ± 4	3.82 ± 0.09	45.0 ± 1.1	0.0057 ± 0.0005
9	0.23 ± 0.31	180 ± 10	65 ± 4	2.85 ± 0.03	43.0 ± 0.9	0.0062 ± 0.0003
10	0.24 ± 0.12	200 ± 10	53 ± 9	3.75 ± 0.02	42.0 ± 0.8	0.0065 ± 0.0012
11	0.22 ± 0.83	130 ± 30	43 ± 9	2.97 ± 0.15	42.0 ± 0.6	0.0043 ± 0.0011
12	0.14 ± 0.54	140 ± 11	46 ± 4	3.08 ± 0.06	36.0 ± 0.7	0.0050 ± 0.0004

## Data Availability

Data available from corresponding author upon request.
